# Inclusion of patients with chronic kidney disease in randomized phase 3 clinical trials in patients with prostate, breast, lung, and colorectal cancer

**DOI:** 10.1002/cam4.5171

**Published:** 2022-09-26

**Authors:** Matthieu Delaye, Adrien Rousseau, Mélanie Try, Christophe Massard, Luca Campedel, Marc Hilmi, Corinne Bagnis

**Affiliations:** ^1^ Department of Medical Oncology, Institut Curie Versailles Saint‐Quentin University Saint‐Cloud France; ^2^ Groupe de Recherche Interdisciplinaire Francophone en Onco‐néphrologie (GRIFON) Paris France; ^3^ Department of Medical Oncology Gustave Roussy Cancer Campus Villejuif France; ^4^ Nephrology department, Bicêtre Hospital APHP Paris‐Saclay University Le Kremlin‐Bicêtre France; ^5^ Department of Medical Oncology, Eugene Marquis Cancer Institute University of Rennes Rennes France; ^6^ Service d'Oncologie CHU Gabriel Montpied, Université Clermont Auvergne Clermont‐Ferrand France; ^7^ Nephrology department, Pitié Salpetrière Hospital APHP Sorbonne University Paris France

**Keywords:** chronic kidney disease, enrollment, onco‐nephrology, phase 3 trials

## Abstract

The objective of this study was to determine the proportion of phase 3 clinical trials investigating a systemic therapy for patients with prostate, breast, lung, or colorectal cancer that excluded patients with Chronic Kidney Disease (CKD) and the exclusion criteria chosen, if any. A search was conducted using the ClinicalTrials.gov database to identify eligible studies. Of the 268 included trials, 185 (69%) had at least one renal exclusion criteria. Of these 185 trials, 116 (63%) had an undefined exclusion criterion. Only disease site was associated with exclusion of patients with CKD in the univariate analysis, but no factors in the multivariate analysis. There are several potential barriers to including patients with CKD in clinical trials. Nevertheless, solutions can be proposed to allow the inclusion of these patients. This would allow them to access to innovative therapeutic strategies, but also allow a better applicability of trial results to this patient population.

## Abstract 2

The majority of clinical trials restrict enrollment of patients with chronic kidney disease (CKD) and the exclusion criterion is undefined in most trials. Patients with CKD have therefore little access to innovation in oncology but solutions can be proposed to allow their inclusion in clinical trials.

Chronic kidney disease (CKD), as defined by the KDIGO Consensus Conference (Glomerular Filtration Rate [GFR] < 60 mL/min/1.73 m^2^ or markers of kidney damage for >3 months) is common in patients treated for cancer.^1^ This is because patients with cancer are at high risk for sepsis, drug‐associated toxicities, tumor lysis syndrome, and other comorbidities that significantly increase the likelihood of developing acute kidney injury.^2^, ^3^ This is also due to the higher risk of cancer in patients on renal replacement therapy (RRT), with kidney transplantation or end‐stage renal disease.^4^ But historically, CKD has been a possible exclusion criterion for clinical trials because of concerns about the risk of altered drug pharmacokinetics and fears of poor prognosis in patients with CKD. As a result, there is little evidence of the efficacy and tolerance of systemic therapies in this population. The objective of this study was to determine the proportion of phase 3 clinical trials investigating a systemic therapy for patients with prostate, breast, lung, or colorectal cancer, the four most common cancers in Western countries, that excluded patients with CKD and the exclusion criteria chosen, if any.

A search was conducted in December 2021, using the ClinicalTrials.gov database to identify eligible studies. The following keywords and filters were applied: “cancer”, “randomized”, “interventional studies”, “studies with results”, exclusion of “not yet recruiting” and “withdrawal”. Of the trials that were obtained, only those investigating a systemic therapy intervention and conducted in prostate cancer, breast cancer, lung cancer, and colorectal cancer were selected (Figure S1). These four cancers were chosen because they are the four most common cancers in Western countries, they affect various populations, and all types of systemic treatments can be used in their management (chemotherapy, hormone therapy, targeted therapy, antibody‐drug conjugate, and immunotherapy). Data collection was arbitrarily restricted to trials with first results posted after March 2013. Screening and data collection were performed by two independent reviewers (Matthieu Delaye and Adrien Rousseau), with discrepancies resolved by a third reviewer (Mélanie Try). All criteria that could exclude a patient because of CKD, including hypertension and ionic disorders that are common in patients with CKD, were collected. An exclusion criterion was judged as undefined if no objective threshold for renal function (serum creatinine or creatinine clearance) was specified (e.g., “adequate organ function”). The statistical analysis was carried out using the R software. Proportions across groups were compared using Pearson's chi‐square tests at a level of significance of *p* = 0.05. The type of funding, the pharmacological class of the investigated drug or control drug, the year of the first inclusion, the inclusion of patients with metastatic disease and whether or not the results were published were the parameters included in the univariate and multivariate analyses.

Of 268 included trials, 125 (47%) were conducted in lung cancer, 81 (30%) in breast cancer, 42 (16%) in prostate cancer and 20 (7%) in colorectal cancer (Table 1) (Table S1). The most frequently studied drug classes were tyrosine kinase inhibitors (*N* = 40; 15%) and monoclonal antibodies (*N* = 33; 12%). Of the 268 included trials, 185 (69%) had at least one renal exclusion criteria. Of these 185 trials, 116 (63%) had an undefined exclusion criterion. The most frequent specified exclusion criteria were uncontrolled hypertension (*N* = 50; 27%) and 1.5‐fold normal elevated serum creatinine (*N* = 31, 17%). Only disease site was associated with exclusion of patients with CKD in the univariate analysis with more frequent renal exclusion criteria in trials conducted in patients with breast or colon cancer than in prostate and lung cancer (*p* = 0.03). However, no factors were found in the multivariate analysis. Besides, only industry funding was associated with providing a specific exclusion criterion in both univariate (*p* < 0.001) and multivariate analysis (OR = 25.3 [95% CI: 6.42–183], *p* < 0.001).

**TABLE 1:** Trial factors associated with exclusion of patients with kidney disease

Trial characteristic	No renal criteria *N* = 83	Renal criteria *N* = 185	*p*‐value^2^	If renal criteria: defined *N* = 69^1^	If renal criteria: undefined *N* = 116^1^	*p*‐value^2^
Disease site			**0.025**			0.2
Lung	43 (52%)	82 (44%)		33 (48%)	49 (42%)	
Prostate	17 (20%)	25 (14%)		11 (16%)	14 (12%)	
Breast	22 (27%)	59 (32%)		16 (23%)	43 (37%)	
Colorectal	1 (1%)	19 (10%)		9 (13%)	10 (9%)	
Industry funding			0.11			**<0.001**
Yes	79 (95%)	165 (89%)		51 (74%)	114 (98%)	
No	4 (5%)	20 (11%)		18 (26%)	2 (2%)	
Investigated drug						0.4
Chemotherapy	1 (1%)	24 (13%)		8 (12%)	16 (14%)	
Combination	33 (40%)	90 (49%)		30 (43%)	60 (52%)	
Hormonotherapy	14 (17%)	17 (9%)		9 (13%)	8 (7%)	
mAb	18 (22%)	15 (8%)		5 (7%)	10 (9%)	
Other drug	5 (6%)	11 (6%)		3 (4%)	8 (7%)	
TKI	12 (14%)	28 (15%)		14 (20%)	14 (12%)	
Control arm			>0.9			0.6
Chemotherapy	32 (39%)	80 (44%)		29 (43%)	51 (44%)	
Combination	12 (15%)	31 (17%)		11 (16%)	20 (17%)	
Hormonotherapy	13 (16%)	24 (13%)		8 (12%)	16 (14%)	
mAb	4 (5%)	7 (4%)		2 (3%)	5 (4%)	
Other drugs	2 (2%)	3 (2%)		0 (0%)	3 (3%)	
Placebo	12 (15%)	24 (13%)		13 (19%)	11 (10%)	
TKI	7 (9%)	15 (8%)		5 (7%)	10 (9%)	
Active surveillance	1 (1%)	1(1%)		1 (1%)	0 (0%)	
Year			0.6			0.5
2004–2010	25 (30%)	57 (31%)		22 (32%)	35 (30%)	
2011–2015	46 (55%)	92 (50%)		31 (45%)	61 (53%)	
2016–2020	12 (14%)	36 (19%)		16 (23%)	20 (17%)	
Patients with MD			>0.9			0.7
Yes	70 (84%)	156 (84%)		59 (86%)	97 (84%)	
No	13 (16%)	29 (16%)		10 (14%)	19 (16%)	
Results published			>0.9			0.3
Yes	63 (76%)	140 (76%)		49 (71%)	91 (78%)	
No	20 (29%)	45 (24%)		25 (22%)	20 (24%)	

Abbreviations: mAB, monoclonal antibody; MD, metastatic disease; TKI, tyrosine kinase inhibitor. ^1^ indicates n (%); ^2^ indicates Pearson's Chi‐squared test; Fisher's exact test. Bold values indicates to statistically significant results.

The majority of phase 3 clinical trials for patients with prostate, breast, lung, or colorectal cancer restricted enrollment of patients with CKD. Moreover, the exclusion criterion was undefined in most trials. However, according to recommendations^5^, ^6^, ^7^ patients with CKD could be routinely included in clinical trials if a method for estimating glomerular filtration rate is used consistently throughout the research process and established dose‐modification strategies are applied. There are several potential barriers to including patients with CKD in clinical trials. Exclusion of patients with CKD may be warranted when CKD is severe and prognosis is limited or when there are concerns about potential nephrotoxicity or adverse effects due to bioaccumulation of renally eliminated drugs. Another argument is that patients with CKD are often multi‐medicated, which increases the risk of drug interactions. However, as shown by our work and previous studies, patients with mild to moderate CKD are also frequently excluded from clinical trials^8^ and often from trials of drug classes for which there is not necessarily a pharmacological basis for renal exclusions (e.g., immunotherapies).^9^ The inclusion of patients with CKD in clinical trials of anticancer drugs is crucial, first because cancer is a major cause of mortality in patients with CKD so that access to innovative therapies is a major issue for these patients, which represent 850 million people worldwide, as for all others.^10^ Second, restrictive eligibility criteria lead to trial results that are not fully applicable to the general patient population, leading to a biased estimate of real‐life treatment benefit. Nevertheless, solutions can be proposed to allow the inclusion of these patients (Table 2).

**TABLE 2:** Common barriers to including patients with CKD in clinical trials

Barrier	Potential solution
Patients with CKD have a worse prognosis	‐ Include patients with stable CKD ‐ Establish criteria for determining the stability of CKD ‐ Stratify on CKD
Pharmacokinetics of drugs is modified due to kidney failure	‐ Choose a method to estimate GFR and use it consistently across the research process ‐ Establish dose‐modification strategies in early phase trials ‐ Perform drug assays ‐ Adapt the GFR thresholds leading to exclusion to the mode of elimination (renal or not) of the drug
Patients with CKD are often multi‐medicated, which increases the risk of drug interactions	‐ Detail the list of contraindicated drugs ‐ Plan a pharmacological follow‐up
The significant burden of comorbidity experienced by patients with CKD makes management complex	‐ Use an interdisciplinary approach

Abbreviations: CKD, chronic kidney disease; GFR, glomerular filtration rate.

Our research was only based on data reported on clinical trial.gov website. The exclusion criteria presented on the website are probably less extensive than those in the complete research protocol. This probably resulted in an overestimated level of undefined exclusion criteria in our study. However, the clinical trial.gov website is often the only platform for physicians to find out about open trials. Thus, the vagueness of exclusion criteria on the website could lead to an over‐exclusion of patients with moderate CKD in current practice. It is important to progressively include patients with CKD in phase 3 trials, even if it means adapting the design. It is also important to unify the exclusion criteria to avoid over‐exclusion of patients with moderate CKD.

## AUTHOR CONTRIBUTIONS

**Matthieu Delaye:** Conceptualization (equal); data curation (equal); writing – original draft (equal); writing – review and editing (equal). **Adrien Rousseau:** Conceptualization (equal); data curation (equal); writing – original draft (equal); writing – review and editing (equal). **Mélanie Try:** Data curation (equal); writing – original draft (equal); writing – review and editing (equal). **Christophe Massard:** Supervision (equal); writing – review and editing (equal). **Luca Campedel:** Conceptualization (equal); writing – original draft (equal). **Marc Hilmi:** Formal analysis (lead); writing – original draft (equal); writing – review and editing (equal). **Corinne Bagnis:** Conceptualization (equal); supervision (equal); writing – review and editing (equal).

## FUNDING INFORMATION

This research did not receive any specific grant from funding agencies in the public, commercial, or not‐for‐profit sectors.

## CONFLICT OF INTEREST

None declared.

## ETHICS APPROVAL

No patient consent was required because the study did not involve patient data. Ethics advice was not required because the data analyzed were public data.

## Supporting information


Table S1

Figure S1
