# Supplementary material for: Inclusion of patients with chronic kidney disease in randomized phase 3 clinical trials in patients with prostate, breast, lung, and colorectal cancer
Source: Cancer Med. 2022 Sep 26;12(3):3172–5. doi: 10.1002/cam4.5171 (PMC9939176; doi:10.1002/cam4.5171)
Supplement: Supplementary file 1 — Table S1 [file CAM4-12-3172-s002.docx]

| **NCT number** | **Tumor** | **Renal exclusion criteria** | **Industrial** | **Experimental** | **Control** | **Year** | **M+ patients** | **Published** |
| --- | --- | --- | --- | --- | --- | --- | --- | --- |
|  |  |  | **funding** | **drug** |  |  | **included** | **results** |
| NCT04633564 | Lung | Hypertension | 1 | combo | combo | 2016-2020 | 1 | 0 |
| NCT03950674 | Lung | Undefined | 1 | combo | Chemo | 2016-2020 | 1 | 1 |
| NCT03875092 | Lung | 0 | 1 | combo | Chemo | 2016-2020 | 1 | 1 |
| NCT03745222 | Lung | Undefined | 1 | combo | combo | 2016-2020 | 0 | 0 |
| NCT03629925 | Lung | Other clearance threshold | 1 | combo | Chemo | 2016-2020 | 1 | 1 |
| NCT03607539 | Lung | Clearance <60; Creatinemia >1,5N | 1 | combo | Chemo | 2016-2020 | 1 | 1 |
| NCT03493854 | Breast | Undefined | 1 | combo | combo | 2016-2020 | 0 | 1 |
| NCT03348904 | Lung | 0 | 1 | combo | Chemo | 2016-2020 | 1 | 0 |
| NCT03329911 | Lung | 0 | 1 | combo | combo | 2016-2020 | 1 | 0 |
| NCT03302234 | Lung | 0 | 1 | mAb | mAb | 2016-2020 | 1 | 1 |
| NCT03296163 | Lung | Creatinemia >1,5N; Other clearance threshold | 1 | combo | combo | 2016-2020 | 1 | 1 |
| NCT03215706 | Lung | 0 | 1 | combo | Chemo | 2016-2020 | 1 | 1 |
| NCT03197935 | Sein | Undefined | 1 | combo | Chemo | 2016-2020 | 0 | 1 |
| NCT03155997 | Breast | Undefined | 1 | combo | hormono | 2016-2020 | 0 | 1 |
| NCT03150875 | Lung | Creatinemia >1,5N; Other clearance threshold | 1 | mAb | Chemo | 2016-2020 | 1 | 0 |
| NCT03125902 | Sein | Undefined | 1 | combo | Chemo | 2016-2020 | 1 | 1 |
| NCT03098030 | Lung | Clearance <30; Creatinemia >1,5N | 1 | combo | Chemo | 2016-2020 | 1 | 0 |
| NCT03085095 | Prostate | Hypertension | 1 | hormono | hormono | 2016-2020 | 1 | 1 |
| NCT03066778 | Lung | Undefined | 1 | combo | Chemo | 2016-2020 | 1 | 1 |
| NCT03061812 | Lung | 0 | 1 | mAb | Chemo | 2016-2020 | 1 | 1 |
| NCT03052608 | Lung | Undefined | 1 | TKI | TKI | 2016-2020 | 1 | 1 |
| NCT03043872 | Lung | Undefined | 1 | combo | Chemo | 2016-2020 | 1 | 1 |
| NCT03033511 | Lung | Undefined | 1 | mAb | placebo | 2016-2020 | 1 | 1 |
| NCT03016312 | Prostate | Undefined | 1 | combo | hormono | 2016-2020 | 1 | 0 |
| NCT02987543 | Prostate | 0 | 1 | other drug | hormono | 2016-2020 | 1 | 1 |
| NCT02965378 | Lung | Other creatinemia threshold; | 0 | TKI | Chemo | 2016-2020 | 1 | 0 |
|  |  | Other clearance threshold |  |  |  |  |  |  |
| NCT02954172 | Lung | Creatinemia >1,5N; Other clearance threshold | 1 | combo | combo | 2016-2020 | 1 | 1 |
| NCT02926638 | Lung | 0 | 0 | combo | TKI | 2016-2020 | 1 | 0 |
| NCT02915744 | Breast | Undefined | 1 | Chemo | Chemo | 2016-2020 | 1 | 1 |
| NCT02899299 | Lung | 0 | 1 | mAb | Chemo | 2016-2020 | 1 | 1 |
| NCT02896855 | Sein | Undefined; Hypertension | 1 | combo | combo | 2016-2020 | 1 | 1 |
| NCT02864394 | Lung | 0 | 1 | mAb | Chemo | 2016-2020 | 1 | 0 |
| NCT02838420 | Lung | Other clearance threshold | 1 | TKI | TKI | 2016-2020 | 1 | 1 |
| NCT02826161 | Lung | Undefined | 1 | Chemo | Chemo | 2016-2020 | 1 | 0 |
| NCT02810457 | Lung | Undefined | 1 | Chemo | Chemo | 2016-2020 | 1 | 1 |
| NCT02785939 | Lung | Other clearance threshold; | 0 | TKI | Chemo | 2016-2020 | 1 | 0 |
|  |  | Other creatinemia threshold |  |  |  |  |  |  |
| NCT02775435 | Lung | Undefined | 1 | combo | Chemo | 2016-2020 | 1 | 1 |
| NCT02768558 | Lung | 0 | 1 | combo | Chemo | 2016-2020 | 0 | 1 |
| NCT02763566 | Breast | Creatinemia >1,5N | 1 | combo | hormono | 2016-2020 | 1 | 1 |
| NCT02763579 | Lung | Undefined | 1 | combo | Chemo | 2016-2020 | 1 | 1 |
| NCT02737501 | Lung | Undefined; Hypertension | 1 | TKI | TKI | 2016-2020 | 1 | 1 |
| NCT02713867 | Lung |  | 1 | mAb | mAb | 2016-2020 | 1 | 0 |
| NCT02677896 | Prostate | Other creatinemia threshold | 1 | hormono | placebo | 2016-2020 | 1 | 1 |
| NCT02657434 | Lung | Clearance <60; Hydroelectrolytic disorder; | 1 | combo | Chemo | 2016-2020 | 1 | 0 |
|  |  | Other clearance threshold |  |  |  |  |  |  |
| NCT02613507 | Lung | 0 | 1 | mAb | Chemo | 2011-2015 | 1 | 1 |
| NCT02604342 | Lung | 0 | 1 | TKI | Chemo | 2011-2015 | 1 | 1 |
| NCT02588261 | Lung | Other clearance threshold; Other creatinemia | 1 | TKI | TKI | 2011-2015 | 1 | 1 |
|  |  | threshold; Hydroelectrolytic disorder |  |  |  |  |  |  |
| NCT02586025 | Breast | Undefined | 1 | combo | combo | 2011-2015 | 0 | 1 |
| NCT02578680 | Lung | Undefined | 1 | combo | Chemo | 2011-2015 | 1 | 1 |
| NCT02574455 | Breast | Clearance <60 | 1 | other drug | Chemo | 2011-2015 | 1 | 1 |
| NCT02566993 | Lung | Undefined | 1 | Chemo | Chemo | 2011-2015 | 1 | 0 |
| NCT02555657 | Breast | Undefined | 1 | mAb | Chemo | 2011-2015 | 1 | 1 |
| NCT02538666 | Lung | 0 | 1 | mAb | placebo | 2011-2015 | 1 | 1 |
| NCT02511106 | Lung | Hypertension; Undefined | 1 | TKI | placebo | 2011-2015 | 0 | 1 |
| NCT02481830 | Lung | 0 | 1 | mAb | Chemo | 2011-2015 | 1 | 0 |
| NCT02472964 | Breast | Creatinemia >1,5N | 1 | combo | combo | 2011-2015 | 1 | 1 |
| NCT02454933 | Lung | Hypertension; Undefined | 1 | combo | TKI | 2011-2015 | 1 | 0 |
| NCT02453282 | Lung | 0 | 1 | mAb | Chemo | 2011-2015 | 1 | 1 |
| NCT02438722 | Lung | Creatinemia >1,5N; | 0 | combo | TKI | 2011-2015 | 1 | 1 |
|  |  | Clearance <60; Hypertension |  |  |  |  |  |  |
| NCT02437318 | Breast | 0 | 1 | combo | hormono | 2011-2015 | 1 | 1 |
| NCT02429427 | Breast | Creatinemia >1,5N; Hypertension | 0 | other drug | placebo | 2011-2015 | 0 | 1 |
| NCT02425891 | Breast | Undefined | 1 | combo | Chemo | 2011-2015 | 1 | 1 |
| NCT02422615 | Breast | Undefined | 1 | combo | hormono | 2011-2015 | 1 | 1 |
| NCT02411448 | Lung | 0 | 1 | combo | TKI | 2011-2015 | 1 | 1 |
| NCT02409342 | Lung | Undefined | 1 | mAb | Chemo | 2011-2015 | 1 | 1 |
| NCT02409355 | Lung | Undefined; Hydroelectrolytic disorder | 1 | mAb | Chemo | 2011-2015 | 1 | 0 |
| NCT02395172 | Lung | Clearance <30 | 1 | mAb | Chemo | 2011-2015 | 1 | 1 |
| NCT02367781 | Lung | Undefined | 1 | combo | Chemo | 2011-2015 | 1 | 1 |
| NCT02367794 | Lung | Undefined | 1 | combo | Chemo | 2011-2015 | 1 | 0 |
| NCT02366143 | Lung | Undefined | 1 | combo | combo | 2011-2015 | 1 | 1 |
| NCT02364999 | Lung | 0 | 1 | combo | combo | 2011-2015 | 1 | 1 |
| NCT02352948 | Lung | 0 | 1 | mAb | combo | 2011-2015 | 1 | 1 |
| NCT02340221 | Breast | Undefined | 1 | combo | hormono | 2011-2015 | 1 | 1 |
| NCT02322281 | Lung | Undefined | 1 | TKI | Chemo | 2011-2015 | 1 | 0 |
| NCT02296125 | Lung | Hypertension | 1 | TKI | TKI | 2011-2015 | 1 | 1 |
| NCT02294461 | Prostate | 0 | 1 | hormono | placebo | 2011-2015 | 1 | 0 |
| NCT02279732 | Lung | 0 | 1 | combo | Chemo | 2011-2015 | 1 | 0 |
| NCT02278120 | Breast | Undefined | 1 | combo | hormono | 2011-2015 | 1 | 1 |
| NCT02272413 | Lung | Undefined | 1 | combo | combo | 2011-2015 | 1 | 0 |
| NCT02264990 | Lung | 0 | 1 | combo | Chemo | 2011-2015 | 1 | 0 |
| NCT02257736 | Prostate | 0 | 1 | hormono | hormono | 2011-2015 | 1 | 1 |
| NCT02246621 | Breast | Undefined | 1 | combo | hormono | 2011-2015 | 1 | 1 |
| NCT02231164 | Lung | 0 | 1 | combo | Chemo | 2011-2015 | 1 | 0 |
| NCT02220894 | Lung | Undefined | 1 | mAb | Chemo | 2011-2015 | 1 | 1 |
| NCT02200614 | Prostate | Other creatinemia threshold; Hypertension | 1 | hormono | placebo | 2016-2020 | 0 | 1 |
| NCT02187744 | Breast | 0 | 1 | combo | combo | 2011-2015 | 0 | 1 |
| NCT02186301 | Lung | Undefined | 1 | TKI | TKI | 2011-2015 | 1 | 0 |
| NCT02175212 | Prostate | 0 | 0 | hormono | hormono | 2011-2015 | 0 | 1 |
| NCT02162667 | Breast | 0 | 1 | mAb | mAb | 2011-2015 | 0 | 1 |
| NCT02152631 | Lung | 0 | 1 | TKI | TKI | 2011-2015 | 1 | 1 |
| NCT02149524 | Breast | Hypertension | 1 | mAb | mAb | 2011-2015 | 0 | 1 |
| NCT02144012 | Breast | Undefined | 1 | other drug | combo | 2011-2015 | 1 | 0 |
| NCT02142738 | Lung | Undefined | 1 | mAb | Chemo | 2011-2015 | 1 | 1 |
| NCT02137837 | Breast | Other creatinemia threshold | 1 | combo | hormono | 2011-2015 | 1 | 0 |
| NCT02134015 | Lung | Undefined; Hypertension | 1 | combo | TKI | 2011-2015 | 1 | 0 |
| NCT02131064 | Breast | 0 | 1 | combo | combo | 2011-2015 | 0 | 1 |
| NCT02125461 | Lung | Hypertension | 1 | mAb | placebo | 2011-2015 | 0 | 1 |
| NCT02115282 | Breast | Other creatinemia threshold | 0 | hormono | hormono | 2011-2015 | 1 | 1 |
| NCT02111577 | Prostate | Creatinemia >1,5N | 1 | combo | Chemo | 2011-2015 | 1 | 0 |
| NCT02107703 | Sein | 0 | 1 | combo | hormono | 2011-2015 | 1 | 1 |
| NCT02085252 | Prostate | 0 | 1 | hormono |  | 2011-2015 | 0 | 0 |
| NCT02075840 | Lung | Undefined | 1 | TKI | TKI | 2011-2015 | 1 | 1 |
| NCT02057666 | Prostate | Creatinemia >1,5N; Hypertension | 1 | other drug | placebo | 2011-2015 | 1 | 0 |
| NCT02054338 | Breast | Undefined | 1 | Chemo | Chemo | 2011-2015 | 1 | 0 |
| NCT02049151 | Lung | Undefined | 1 | Chemo | placebo | 2011-2015 | 0 | 0 |
| NCT02043678 | Prostate | 0 | 1 | combo | hormono | 2011-2015 | 1 | 1 |
| NCT02041533 | Lung | 0 | 1 | mAb | Chemo | 2011-2015 | 1 | 1 |
| NCT02027428 | Lung | Other creatinemia threshold | 1 | Chemo | Chemo | 2011-2015 | 1 | 1 |
| NCT02008227 | Lung | 0 | 1 | mAb | Chemo | 2011-2015 | 1 | 1 |
| NCT02003924 | Prostate | Other creatinemia threshold | 1 | hormono | placebo | 2011-2015 | 0 | 1 |
| NCT02000622 | Breast | Undefined | 1 | other drug | Chemo | 2011-2015 | 1 | 1 |
| NCT01998906 | Breast | 0 | 1 | combo | Chemo | 2011-2015 | 0 | 0 |
| NCT01989676 | Breast | 0 | 1 | combo | combo | 2011-2015 | 1 | 1 |
| NCT01966003 | Lung | 0 | 1 | combo | combo | 2011-2015 | 1 | 1 |
| NCT01966471 | Breast | Hypertension; Undefined | 1 | combo | combo | 2011-2015 | 0 | 0 |
| NCT01958021 | Breast | Hypertension | 1 | combo | hormono | 2011-2015 | 1 | 1 |
| NCT01953003 | Breast | Other clearance threshold | 1 | Chemo | Chemo | 2011-2015 | 1 | 0 |
| NCT01949337 | Prostate | Hypertension; Other creatinemia threshold | 1 | hormono | hormono | 2011-2015 | 1 | 0 |
| NCT01945775 | Breast | 0 | 1 | other drug | Chemo | 2011-2015 | 1 | 1 |
| NCT01946204 | Prostate | Undefined | 1 | hormono | placebo | 2011-2015 | 0 | 1 |
| NCT01942135 | Breast | Undefined | 1 | combo | hormono | 2011-2015 | 1 | 1 |
| NCT01933932 | poumon | 0 | 1 | combo | Chemo | 2011-2015 | 1 | 1 |
| NCT01905592 | Breast | Undefined | 1 | other drug | Chemo | 2011-2015 | 1 | 0 |
| NCT01905657 | Lung | 0 | 1 | mAb | Chemo | 2011-2015 | 1 | 1 |
| NCT01901146 | Breast | Undefined; Hypertension | 1 | combo | combo | 2011-2015 | 0 | 1 |
| NCT01881230 | Breast | Clearance <60 | 1 | Chemo | Chemo | 2011-2015 | 1 | 1 |
| NCT01875367 | Breast | Undefined; Hypertension | 0 | mAb | mAb | 2011-2015 | 1 | 0 |
| NCT01828099 | Lung | 0 | 1 | TKI | Chemo | 2011-2015 | 1 | 1 |
| NCT01828112 | Lung | 0 | 1 | TKI | Chemo | 2011-2015 | 1 | 1 |
| NCT01808573 | Breast | 0 | 1 | combo | combo | 2011-2015 | 1 | 1 |
| NCT01798485 | Lung | Undefined | 1 | combo | Chemo | 2011-2015 | 1 | 0 |
| NCT01774721 | Lung | Undefined | 1 | TKI | TKI | 2011-2015 | 1 | 1 |
| NCT01772472 | Breast | Undefined | 1 | other drug | mAb | 2011-2015 | 0 | 1 |
| NCT01763645 | Lung | Other creatinemia threshold; Hypertension; | 1 | combo | combo | 2011-2015 | 1 | 0 |
| NCT01764022 | Breast | Hypertension; Creatinemia >1,5N | 1 | combo | combo | 2011-2015 | 1 | 1 |
| NCT01740427 | Breast | Undefined | 1 | combo | hormono | 2011-2015 | 1 | 1 |
| NCT01715285 | Prostate | Undefined | 1 | hormono | hormono | 2011-2015 | 1 | 1 |
| NCT01695135 | Prostate | Hypertension | 1 | hormono | placebo | 2011-2015 | 1 | 0 |
| NCT01673867 | Lung | 0 | 1 | mAb | Chemo | 2011-2015 | 1 | 1 |
| NCT01663727 | Breast | Undefined | 1 | combo | Chemo | 2011-2015 | 1 | 1 |
| NCT01652469 | Lung | Other clearance threshold | 1 | TKI | Chemo | 2011-2015 | 1 | 1 |
| NCT01644890 | Breast | 0 | 1 | other drug | Chemo | 2011-2015 | 1 | 1 |
| NCT01642004 | Lung | 0 | 1 | mAb | Chemo | 2011-2015 | 1 | 1 |
| NCT01639001 | Lung | Undefined | 1 | TKI | Chemo | 2011-2015 | 1 | 1 |
| NCT01633060 | Breast | Undefined | 1 | combo | hormono | 2011-2015 | 1 | 1 |
| NCT01610284 | Breast | Undefined | 1 | combo | hormono | 2011-2015 | 1 | 1 |
| NCT01605227 | Prostate | Undefined | 1 | TKI | other drug | 2011-2015 | 1 | 1 |
| NCT01602380 | Breast | 0 | 1 | hormono | hormono | 2011-2015 | 1 | 1 |
| NCT01572727 | Breast | Undefined | 1 | combo | Chemo | 2011-2015 | 1 | 0 |
| NCT01566721 | Breast | Undefined; Hypertension | 1 | mAb | mAb | 2011-2015 | 0 | 1 |
| NCT01546623 | Prostate | Creatinemia >1,5N | 1 | hormono | hormono | 2011-2015 | 1 | 0 |
| NCT01546649 | Breast | Creatinemia >1,5N | 1 | hormono | hormono | 2011-2015 | 0 | 0 |
| NCT01544179 | Lung | Undefined | 1 | combo | Chemo | 2011-2015 | 1 | 1 |
| NCT01522443 | Prostate | Undefined | 1 | TKI | Chemo | 2011-2015 | 1 | 1 |
| NCT01523587 | Lung | Undefined | 1 | TKI | TKI | 2011-2015 | 1 | 1 |
| NCT01492101 | Breast | Undefined | 1 | Chemo | Chemo | 2011-2015 | 1 | 1 |
| NCT01454934 | Lung | Undefined | 1 | Chemo | Chemo | 2011-2015 | 1 | 1 |
| NCT01450761 | Lung | 0 | 1 | combo | Chemo | 2011-2015 | 1 | 1 |
| NCT01419197 | Breast | Undefined | 1 | other drug | other drug | 2011-2015 | 1 | 1 |
| NCT01404260 | Lung | Undefined | 0 | combo | Chemo | 2011-2015 | 1 | 0 |
| NCT01360554 | Lung | Undefined | 1 | TKI | TKI | 2011-2015 | 1 | 1 |
| NCT01358877 | Breast | Undefined | 1 | combo | combo | 2011-2015 | 0 | 1 |
| NCT01351415 | Lung | Undefined | 1 | combo | combo | 2011-2015 | 1 | 1 |
| NCT01342965 | Lung | 0 | 1 | TKI | Chemo | 2011-2015 | 1 | 1 |
| NCT01328951 | Lung | 0 | 1 | combo | TKI | 2011-2015 | 1 | 0 |
| NCT01322490 | Prostate | 0 | 1 | combo | placebo | 2011-2015 | 1 | 1 |
| NCT01313273 | Prostate | 0 | 1 | hormono | hormono | 2011-2015 | 1 | 0 |
| NCT01308567 | Prostate | Undefined | 1 | Chemo | Chemo | 2011-2015 | 1 | 1 |
| NCT01308580 | Prostate | Hypertension | 1 | Chemo | Chemo | 2011-2015 | 1 | 1 |
| NCT01300351 | Breast | 0 | 1 | hormono | hormono | 2011-2015 | 1 | 0 |
| NCT01285609 | Lung | 0 | 1 | combo | Chemo | 2011-2015 | 1 | 0 |
| NCT01275677 | Breast | Other creatinemia threshold; Hypertension | 0 | combo | Chemo | 2011-2015 | 0 | 1 |
| NCT01250379 | Breast | Undefined | 1 | combo | Chemo | 2004-2010 | 1 | 1 |
| NCT01244191 | Lung | Creatinemia >1,5N; Clearance <60; | 1 | TKI | TKI | 2004-2010 | 1 | 1 |
|  |  | Hypertension |  |  |  |  |  |  |
| NCT01237327 | Breast | 0 | 1 | hormono | hormono | 2004-2010 | 1 | 0 |
| NCT01234337 | Breast | Undefined | 1 | combo | Chemo | 2004-2010 | 1 | 1 |
| NCT01212991 | Prostate | 0 | 1 | hormono | placebo | 2004-2010 | 1 | 1 |
| NCT01193244 | Prostate | 0 | 1 | hormono | other drug | 2004-2010 | 1 | 1 |
| NCT01193257 | Prostate | Undefined | 1 | hormono | other drug | 2004-2010 | 1 | 1 |
| NCT01183858 | Lung | 0 | 1 | TKI | TKI | 2004-2010 | 1 | 0 |
| NCT01168973 | Lung | Creatinemia >1,5N; Hypertension | 1 | combo | Chemo | 2004-2010 | 1 | 1 |
|  |  | Other clearance threshold; Proteinuria |  |  |  |  |  |  |
| NCT01160211 | Breast | 0 | 1 | combo | combo | 2004-2010 | 1 | 1 |
| NCT01154140 | Lung | 0 | 1 | TKI | Chemo | 2011-2015 | 1 | 1 |
| NCT01133704 | Prostate | Undefined | 1 | other drug | placebo | 2004-2010 | 1 | 1 |
| NCT01125566 | Breast | Hypertension; Undefined | 1 | combo | combo | 2004-2010 | 1 | 1 |
| NCT01121393 | Lung | Clearance <60; Creatinemia >1,5N | 1 | TKI | Chemo | 2004-2010 | 1 | 1 |
| NCT01120184 | Breast | Undefined | 1 | combo | combo | 2004-2010 | 1 | 1 |
| NCT01107626 | Lung | Other creatinemia threshold; | 0 | combo | combo | 2004-2010 | 1 | 1 |
|  |  | Clearance <60; Proteinuria; Hypertension |  |  |  |  |  |  |
| NCT01095003 | Breast | Undefined | 1 | Chemo | Chemo | 2004-2010 | 1 | 1 |
| NCT01091168 | Breast | Undefined | 1 | Chemo | Chemo | 2004-2010 | 1 | 1 |
| NCT01085136 | Lung | Hypertension; Creatinemia >1,5N; | 1 | combo | Chemo | 2004-2010 | 1 | 1 |
|  |  | Other clearance threshold |  |  |  |  |  |  |
| NCT01073865 | Breast | 0 | 1 | hormono | hormono | 2004-2010 | 1 | 0 |
| NCT01057810 | Prostate | 0 | 1 | mAb | placebo | 2004-2010 | 1 | 1 |
| NCT01041781 | Lung | Other clearance threshold | 0 | combo | Chemo | 2004-2010 | 1 | 1 |
| NCT01040780 | Lung | 0 | 1 | TKI | TKI | 2004-2010 | 1 | 1 |
| NCT01026142 | Sein | Hypertension; Undefined | 1 | combo | combo | 2004-2010 | 1 | 0 |
| NCT01017874 | Breast | 0 | 1 | combo | TKI | 2004-2010 | 1 | 1 |
| NCT01015443 | Lung | Undefined | 1 | combo | placebo | 2004-2010 | 0 | 1 |
| NCT01007942 | Breast | 0 | 1 | combo | combo | 2004-2010 | 1 | 1 |
| NCT01005680 | Lung | Undefined | 1 | Chemo | Chemo | 2004-2010 | 1 | 0 |
| NCT01000025 | Lung | Creatinemia >1,5N | 1 | TKI | placebo | 2004-2010 | 1 | 1 |
| NCT00988208 | Prostate | Undefined | 1 | combo | Chemo | 2004-2010 | 1 | 1 |
| NCT00981058 | Lung | Undefined | 1 | combo | Chemo | 2004-2010 | 1 | 1 |
| NCT00982111 | Lung | Undefined | 1 | combo | Chemo | 2004-2010 | 1 | 1 |
| NCT00976456 | Lung | 0 | 1 | combo | combo | 2004-2010 | 1 | 0 |
| NCT00974311 | Prostate | Undefined | 1 | hormono | placebo | 2004-2010 | 1 | 1 |
| NCT00968968 | Breast | Undefined | 1 | combo | mAb | 2004-2010 | 1 | 0 |
| NCT00961415 | Lung | Undefined | 1 | combo | combo | 2004-2010 | 1 | 1 |
| NCT00950300 | Sein | Undefined | 1 | combo | combo | 2004-2010 | 0 | 1 |
| NCT00949650 | Lung | Undefined | 1 | TKI | Chemo | 2004-2010 | 1 | 1 |
| NCT00948675 | Lung | Undefined; Hypertension | 1 | combo | Chemo | 2004-2010 | 1 | 1 |
| NCT00946712 | Lung | Other creatinemia threshold; Hypertension; | 0 | combo | combo | 2004-2010 | 1 | 1 |
|  |  | Other clearance threshold; Proteinuria |  |  |  |  |  |  |
| NCT00946920 | Prostate | Undefined | 1 | hormono | hormono | 2004-2010 | 1 | 1 |
| NCT00932893 | Lung | 0 | 1 | TKI | Chemo | 2004-2010 | 1 | 1 |
| NCT00928434 | Prostate | Undefined | 1 | hormono | hormono | 2004-2010 | 0 | 1 |
| NCT00929240 | Breast | 0 | 1 | combo | mAb | 2004-2010 | 1 | 1 |
| NCT00925548 | Breast | Undefined | 1 | combo | hormono | 2004-2010 | 1 | 0 |
| NCT00887198 | Prostate | 0 | 1 | hormono | placebo | 2004-2010 | 1 | 1 |
| NCT00883779 | Lung | 0 | 1 | combo | Chemo | 2004-2010 | 1 | 1 |
| NCT00884273 | Prostate | 0 | 1 | hormono | hormono | 2004-2010 | 0 | 1 |
| NCT00878709 | Breast | 0 | 1 | TKI | placebo | 2004-2010 | 0 | 1 |
| NCT00876395 | Breast | Undefined; Hypertension | 1 | combo | combo | 2004-2010 | 1 | 1 |
| NCT00863512 | Lung | Creatinemia >1,5N | 0 | Chemo |  | 2004-2010 | 0 | 0 |
| NCT00863655 | Breast | 0 | 1 | combo | hormono | 2004-2010 | 1 | 1 |
| NCT00863746 | Lung | Creatinemia >1,5N; Hypertension; | 1 | TKI | placebo | 2004-2010 | 1 | 0 |
|  |  | Other clearance threshold |  |  |  |  |  |  |
| NCT00861614 | Prostate | 0 | 1 | mAb | placebo | 2004-2010 | 1 | 1 |
| NCT00833248 | Prostate | Undefined | 1 | hormono | hormono | 2004-2010 | 0 | 1 |
| NCT00831233 | Prostate | Undefined | 1 | hormono | hormono | 2004-2010 | 0 | 1 |
| NCT00829166 | Breast | Undefined | 1 | other drug | combo | 2004-2010 | 1 | 1 |
| NCT00820755 | Lung | Clearance <60 | 1 | combo | mAb | 2004-2010 | 1 | 1 |
| NCT00806819 | Lung | Hypertension; Undefined | 1 | combo | Chemo | 2004-2010 | 1 | 0 |
| NCT00805194 | Lung | Undefined | 1 | combo | Chemo | 2004-2010 | 1 | 1 |
| NCT00795340 | Lung | Other clearance threshold; Hypertension | 0 | combo | Chemo | 2004-2010 | 1 | 1 |
| NCT00789581 | Breast | Creatinemia >1,5N | 1 | Chemo | Chemo | 2004-2010 | 0 | 1 |
| NCT00789373 | Lung | Undefined | 1 | Chemo | Chemo | 2004-2010 | 1 | 1 |
| NCT00785291 | Breast | Hypertension; Proteinuria; | 0 | combo | combo | 2004-2010 | 1 | 1 |
|  |  | Other creatinemia threshold |  |  |  |  |  |  |
| NCT00779402 | Prostate | 0 | 1 | other drug | placebo | 2004-2010 | 0 | 1 |
| NCT00770809 | Breast | 0 | 0 | combo | combo | 2004-2010 | 0 | 1 |
| NCT00762034 | Lung | Undefined; Hypertension | 1 | combo | combo | 2004-2010 | 1 | 1 |
| NCT00754845 | Breast | 0 | 0 | hormono | placebo | 2004-2010 | 0 | 1 |
| NCT00744497 | Prostate | Creatinemia >1,5N | 1 | combo | Chemo | 2004-2010 | 1 | 1 |
| NCT00738881 | Lung | Other clearance threshold; Hypertension | 0 | TKI | Chemo | 2004-2010 | 1 | 0 |
| NCT00703326 | Breast | Creatinemia >1,5N; Hypertension; | 1 | combo | Chemo | 2004-2010 | 1 | 1 |
|  |  | Other clearance threshold; Proteinuria |  |  |  |  |  |  |
| NCT00699751 | Prostate | 0 | 1 | other drug | placebo | 2004-2010 | 1 | 1 |
| NCT00693992 | Lung | Hypertension; Other creatinemia threshold | 0 | TKI | placebo | 2004-2010 | 1 | 1 |
| NCT00688740 | Breast | 0 | 1 | Chemo | Chemo | 2004-2010 | 0 | 1 |
| NCT00686959 | Lung | Undefined | 1 | Chemo | Chemo | 2004-2010 | 0 | 1 |
| NCT00676650 | Prostate | 0 | 1 | TKI | other drug | 2004-2010 | 1 | 1 |
| NCT01661270 | Colorectal | Undefined ; Proteinuria ; Hypertension | 1 | Combo | Chemo | 2011-2015 | 1 | 1 |
| NCT01607957 | Colorectal | Undefined | 1 | Chemo | Placebo | 2011-2015 | 1 | 1 |
| NCT01412957 | Colorectal | Undefined | 1 | mAb | Placebo | 2011-2015 | 1 | 1 |
| NCT01279681 | Colorectal | Creatinemia >1,5N ; Proteinuria ; Clearance <60 ; | 0 | Combo | Combo | 2011-2015 | 1 | 1 |
|  |  | Hypertension |  |  |  |  |  |  |
| NCT01228734 | Colorectal | Creatinemia >1,5N ; Hypertension | 1 | Combo | Chemo | 2004-2010 | 1 | 1 |
| NCT01189227 | Colorectal | Other creatinemia threshold ; Clearance <30 ; | 0 | Chemo | Chemo | 2004-2010 | 1 | 0 |
|  |  | Hypertension |  |  |  |  |  |  |
| NCT01183780 | Colorectal | Undefined | 1 | Combo | Chemo | 2004-2010 | 1 | 1 |
| NCT01150045 | Colorectal | Hypertension ; Creatinemia >1,5N | 0 | Combo | Chemo | 2004-2010 | 0 | 1 |
| NCT01103323 | Colorectal | Undefined ; Proteinuria | 1 | TKI | Placebo | 2004-2010 | 1 | 1 |
| NCT01099449 | Colorectal | Creatinemia >1,5N | 0 | Chemo | Chemo | 2004-2010 | 1 | 1 |
| NCT01001377 | Colorectal | Undefined | 1 | mAb | mAb | 2004-2010 | 1 | 1 |
| NCT03288987 | Colorectal | Proteinuria | 1 | Combo | Combo | 2016-2020 | 1 | 1 |
| NCT02928224 | Colorectal | Hypertension ; Undefined | 1 | Combo | Combo | 2016-2020 | 1 | 1 |
| NCT02788279 | Colorectal | Hypertension ; Undefined | 1 | Combo | TKI | 2016-2020 | 1 | 1 |
| NCT02149108 | Colorectal | 0 | 1 | TKI | placebo | 2011-2015 | 1 | 1 |
| NCT01955837 | Colorectal | Undefined | 1 | Chemo | placebo | 2011-2015 | 1 | 1 |
| NCT01939223 | Colorectal | Creatinemia >1,5N ;  Clearance <30 ; | 1 | TKI | placebo | 2011-2015 | 1 | 0 |
|  |  | Hypertension |  |  |  |  |  |  |
| NCT01830621 | Colorectal | Creatinemia >1,5N ; Other clearance threshold | 1 | TKI | placebo | 2011-2015 | 1 | 1 |
| NCT01767857 | Colorectal | Creatinemia >1,5N | 1 | mAb | placebo | 2011-2015 | 1 | 1 |
| NCT01767857 | Colorectal | Undefined | 1 | other | Chemo | 2011-2015 | 1 | 1 |

**Supplementary Table 1: Clinical trials included in the study**

1 : Yes, 0 : No, M+ : metastatic, Chemo : Chemotherapy, Combo : combination of treatments from different classes, mAb : monoclonal antibody, hormono : hormonotherapy, TKI : Tyrosine Kinase Inhibitor
