# Supplementary material for: Inclusion of patients with chronic kidney disease in randomized phase 3 clinical trials in patients with prostate, breast, lung, and colorectal cancer
Source: Cancer Med. 2022 Sep 26;12(3):3172–5. doi: 10.1002/cam4.5171 (PMC9939176; doi:10.1002/cam4.5171)
Supplement: Supplementary file 2 — Figure S1 [file CAM4-12-3172-s001.docx]

**Supplementary Figure 1 : Flowchart**

**Identification of studies via databases and registers**

Records identified from clinicaltrial.org (n = 904)

**Identification**

Reports excluded:

Other Cancer (n = 452)

No systemic drug (n = 35)

Other disease (n = 149)

Records screened

(n = 904)

**Screening**

**Included**

Reports of included studies

(n = 268)
